# 402. Multidisciplinary Management Model for Multisystem Inflammatory Syndrome in Children: A Comprehensive Approach for Improved Patient Outcomes

**DOI:** 10.1093/ofid/ofad500.472

**Published:** 2023-11-27

**Authors:** Sarah Gillen, Stacie Knutson, Jonathan Strutt, Gwenyth Fischer, Bazak Sharon, Alexander A Boucher, Marie E Steiner, Bryce A Binstadt, Jordan Marmet, Kate Sadak

**Affiliations:** University of Minnesota, Minnepolis, Minnesota; University of Minnesota, MHealth Fairview Masonic Children's Hospital, Minneapolis, Minnesota; University of Minnesota, Minnepolis, Minnesota; University of Minnesota, Minnepolis, Minnesota; University of Minnesota, Minnepolis, Minnesota; University of Minnesota, Minnepolis, Minnesota; University of Minnesota, Minnepolis, Minnesota; University of Minnesota, Minnepolis, Minnesota; University of Minnesota Medical School, Minneapolis, Minnesota; MHealth Fairview, Minneapolis, Minnesota

## Abstract

**Background:**

In the spring 2020, Multisystem Inflammatory Syndrome in Children (MIS-C) emerged as a serious threat to children. As case definitions and guidelines were being developed, pediatricians worldwide faced the challenge of identifying and managing this novel condition. Here, we describe our institution's approach to management of MIS-C and describe the outcomes of our patient cohort.

**Methods:**

At the University of Minnesota, a team of specialists in infectious diseases, cardiology, hematology, rheumatology, intensive care, hospital medicine, emergency medicine, and primary care was formed to address emerging concerns. The group collaborated to draft clinical guidelines that were shared locally with clinics and emergency rooms [Figure 1]. The team established multidisciplinary rounds (“MIS-C calls“) which were held remotely via Zoom to facilitate acute care coordination and long-term management plans to be discussed among primary providers and specialists.

**Figure 1**

**Figure:**
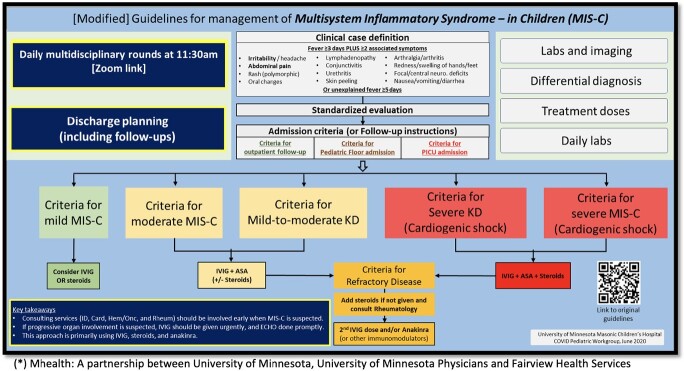

**Results:**

Between May 2020 and January 2022, thirty-five children were admitted to the University of Minnesota Masonic Children’s Hospital and diagnosed with MIS-C [Figure 2]. Complications included myocarditis, coronary aneurysm, coagulopathy, acute kidney injury, and shock. Seventeen children required PICU admission. All patients received standardized and comprehensive care, including MIS-C calls, while hospitalized and coordinated ambulatory care after discharge. There were no deaths or long-term cardiovascular morbidity noted through longitudinal follow-up.

**Figure 2**

**Figure:**
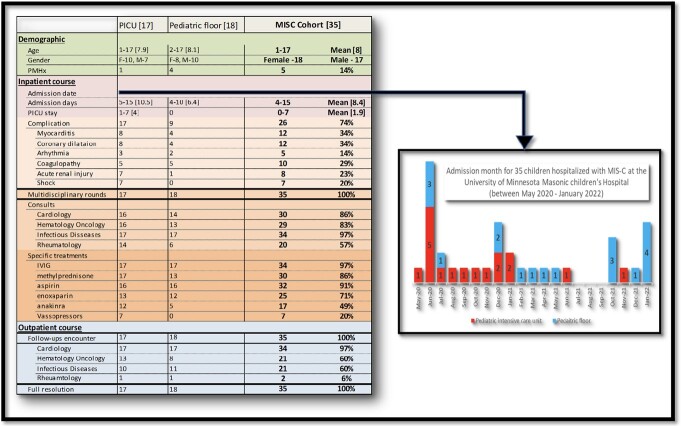

Demographic, clinical presentation, hospital course, and out-patient follow-up for a cohort of 35 children hospitalized with MIS-C at the University of Minnesota Masonic Children’s Hospital, between May 2020 and January 2022.

**Conclusion:**

Our institution's multidisciplinary MIS-C model involved a collaborative effort among specialists from various disciplines. By designing and implementing clinical guidelines that were locally disseminated, and establishing daily multidisciplinary rounds, we were able to effectively identify MIS-C cases across our region and provide effective management at our hospital and clinics. Outcomes were favorable without mortality or significant morbidity as all patients fully recovered without any serious long-term sequelae. These findings highlight the importance of a multidisciplinary, comprehensive, and coordinated approach that is well communicated throughout clinical settings, in managing rapidly emerging medical crises.

**Disclosures:**

**Gwenyth Fischer, MD**, Abiomed: Advisor/Consultant **Alexander A. Boucher, MD**, CSL Behring: Grant/Research Support **Marie E. Steiner, MD**, Medtronic: Teaching|Octapharma: Advisor/Consultant **Bryce A. Binstadt, MD, PhD**, Sobi: Grant/Research Support

